# Perivascular Adipose Tissue Harbors Atheroprotective IgM-Producing B Cells

**DOI:** 10.3389/fphys.2017.00719

**Published:** 2017-09-22

**Authors:** Prasad Srikakulapu, Aditi Upadhye, Sam M. Rosenfeld, Melissa A. Marshall, Chantel McSkimming, Alexandra W. Hickman, Ileana S. Mauldin, Gorav Ailawadi, M. Beatriz S. Lopes, Angela M. Taylor, Coleen A. McNamara

**Affiliations:** ^1^Cardiovascular Research Center, University of Virginia Charlottesville, VA, United States; ^2^Department of Surgery, University of Virginia Charlottesville, VA, United States; ^3^Department of Pathology and Neurological Surgery, University of Virginia Charlottesville, VA, United States; ^4^Department of Medicine, Division of Cardiovascular Medicine, University of Virginia Charlottesville, VA, United States

**Keywords:** B cells, IgM, atherosclerosis, inflammation, perivascular adipose tissue, fat associated lymphoid clusters

## Abstract

Adipose tissue surrounding major arteries (Perivascular adipose tissue or PVAT) has long been thought to exist to provide vessel support and insulation. Emerging evidence suggests that PVAT regulates artery physiology and pathology, such as, promoting atherosclerosis development through local production of inflammatory cytokines. Yet the immune subtypes in PVAT that regulate inflammation are poorly characterized. B cells have emerged as important immune cells in the regulation of visceral adipose tissue inflammation and atherosclerosis. B cell-mediated effects on atherosclerosis are subset-dependent with B-1 cells attenuating and B-2 cells aggravating atherosclerosis. While mechanisms whereby B-2 cells aggravate atherosclerosis are less clear, production of immunoglobulin type M (IgM) antibodies is thought to be a major mechanism whereby B-1 cells limit atherosclerosis development. B-1 cell-derived IgM to oxidation specific epitopes (OSE) on low density lipoproteins (LDL) blocks oxidized LDL-induced inflammatory cytokine production and foam cell formation. However, whether PVAT contains B-1 cells and whether atheroprotective IgM is produced in PVAT is unknown. Results of the present study provide clear evidence that the majority of B cells in and around the aorta are derived from PVAT. Interestingly, a large proportion of these B cells belong to the B-1 subset with the B-1/B-2 ratio being 10-fold higher in PVAT relative to spleen and bone marrow. Moreover, PVAT contains significantly greater numbers of IgM secreting cells than the aorta. ApoE^−/−^ mice with B cell-specific knockout of the gene encoding the helix-loop-helix factor Id3, known to have attenuated diet-induced atherosclerosis, have increased numbers of B-1b cells and increased IgM secreting cells in PVAT relative to littermate controls. Immunostaining of PVAT on human coronary arteries identified fat associated lymphoid clusters (FALCs) harboring high numbers of B cells, and flow cytometry demonstrated the presence of T cells and B cells including B-1 cells. Taken together, these results provide evidence that murine and human PVAT harbor B-1 cells and suggest that local IgM production may serve to provide atheroprotection.

## Introduction

Atherosclerosis, a chronic inflammatory disease of arteries, is the major underlying cause of cardiovascular disease (CVD). Atherosclerosis develops when low density lipoprotein (LDL) enters the artery wall, becomes oxidized. Products of oxidized lipids are highly reactive and modify self-molecules, thereby generating structural neo-epitopes that are recognized by receptors of the immune system, including scavenger receptors on macrophages leading to foam cell formation. These neo-epitopes are termed oxidation specific epitopes (OSEs) and represent a common set of epitopes present on various oxidatively modified self-proteins and lipids (Chou et al., 2008; Miller et al., 2011). OSEs, including oxidized phospholipids (OxPLs) and malondialdehyde (MDA)-modified amino groups, have also been documented on the surface of apoptotic cells and microvesicles (Chang et al., 2004; Miller et al., 2011; Tsiantoulas et al., 2015). Oxidized LDL (OxLDL) and foam cells promote inflammatory cytokine production and endothelial cell adhesion molecule expression, leading to recruitment of inflammatory cells such as, monocytes, T cells, natural killer cells, natural killer T cells, and dendritic cells into the intima, fueling lesion formation (Hansson and Hermansson, 2011; Wigren et al., 2012; Binder et al., 2016).

B cells have emerged as important immune cells in the regulation of atherosclerosis. B cells regulate immune responses by secreting antibodies and cytokines (Srikakulapu and McNamara, 2017) and can be divided into B-1 and B-2 subtypes. B-2 cells are abundant in secondary lymphoid organs (SLOs), play a major role in adaptive immune responses and are thought to promote atherosclerosis. In contrast, B-1 cells, the major source for natural IgM secretion in the body (Corte-Real et al., 2009; Holodick et al., 2010; Choi et al., 2012), produce IgM to OSE on OxLDL and provide innate immune protection from diet-induced atherosclerosis in mice (Kyaw et al., 2011; Rosenfeld et al., 2015).

Obesity is an important risk factor for CVD. Notably, obesity-induced metabolic dysfunction in adipose tissue depots is linked to inflammation (Fuster et al., 2016). Macrophages infiltrate expanding adipose tissue in response to chemokines produced by adipose tissue in high fat diet-fed mice (Amano et al., 2014; Bai and Sun, 2015; Kaplan et al., 2015), and promote further inflammation through the production of inflammatory cytokines. B-1 and B-2 cells are also present in murine and human adipose tissues and have recently been found to produce immunoglobulins within viceral and subcutanoues adipose tissues, suggesting a mechanism whereby they may participate in regulation of adipose tissue inflammation (Winer et al., 2011; Harmon et al., 2016). B-1 cells in visceral adipose tissue attenuated high fat diet-induced macrophage production of inflammatory cytokines such as, tumor necrosis factor-alpha (TNFα) and monocyte chemoattractant protein-1 (MCP-1) (Harmon et al., 2016). We identified Inhibitor of differentiation-3 (Id3), a basic helix-loop-helix (bHLH) protein that inhibits E-proteins binding to DNA to regulate transcription as an inhibitor of B-1 cell number and IgM production in visceral adipose tissue (Harmon et al., 2016).

In addition to subcutaneous and visceral adipose tissue, adipose tissue surrounds major blood vessels and is called perivascular adipose tissue (PVAT). Historically, PVAT has been thought to function in blood vessel support. However, emerging literature support a role for PVAT in other biological processes such as, maintaining vasomotar tone (Nosalski and Guzik, 2017). Moreover, PVAT contains immune cells such as, macrophages, T cell subsets, NK cells, and dendritic cells (Guzik et al., 2007; Chan et al., 2012; Moore et al., 2015; Wensveen et al., 2015; Mikolajczyk et al., 2016), produces both pro-inflammatory and anti-inflammatory cytokines and has been shown to regulate atherosclerosis in mice (Gustafson, 2010; Rajsheker et al., 2010; Manka et al., 2014). Recently, Moro K et al, discovered lymphoid aggregates in the adipose tissue of normal healthy mice, termed fat associated lymphoid clusters (FALCs) (Moro et al., 2010). Recently, Newland SA et al, have shown the presence of FALCs in peri-aortic adipose tissue of 80 weeks old ApoE^−/−^ mice and these FALCs harbored high numbers of B cells and T cells (Newland et al., 2017). Consistent with these murine findings, PVAT adjacent to human atherosclerotic arteries is more inflamed than PVAT adjacent to normal arteries (Henrichot et al., 2005). Yet, whether FALCs are available in human PVAT near coronary arteries is unknown. We hypothesized that B-1 cells are present in mouse and human PVAT and produce anti-inflammatory IgM in this depot. We further hypothesized that Id3 is an important regulatory factor for B-1 accumulation in PVAT.

In this study, we characterized B cell composition in murine and human PVAT. We provide the first evidence that PVAT contains the majority of artery-associated B cells at homeostasis and in response to Western diet (WD). Moreover, in comparison to other B cell niches, PVAT contains an enriched population of B-1 cell subsets that produce IgM to OSE. Furthermore, we identified Id3 as a critical transcription factor regulating PVAT B-1b cell number. Taken together with previous studies implicating B-1 cells in inhibiting inflammation and providing atheroprotection (Rosenfeld et al., 2015; Harmon et al., 2016), these results suggest that mechanisms to boost B-1b cell antibody production in the PVAT may have important therapeutic implications in early prevention of atherosclerosis.

## Materials and methods

### Animals

All animal protocols were approved by the Animal Care and Use Committee at the University of Virginia. Apolipoprotein E deficient (ApoE^−/−^) mice were purchased from Jackson Laboratory and maintained in our animal facility (University of Virginia). Id3^fl/fl^ mice were a generous gift from Dr. Yuan Zhang (Duke University). CD19^Cre/+^ mice were provided by Timothy Bender (University of Virginia). Id3^fl/fl^ mice were bred to the ApoE^−/−^ line and then with CD19^Cre/+^ mice to develop B cell specific Id3 knockouts and littermate controls (ApoE^−/−^Id3^WT^: ApoE^−/−^.CD19^+/+^.Id3^fl/fl^ and ApoE^−/−^Id3^BKO^: ApoE^−/−^.CD19^Cre/+^.Id3^fl/fl^) as previously described (Perry et al., 2013). All purchased mice were on C57BL/6J background and those bred were backcrossed to C57BL/6J mice for 10 generations. All mice were given water *ad libitum* and standard chow diet (Tekland, 7012). Mice were euthanized with CO_2_ inhalation. Young (8–10 weeks) male mice were used for all experiments except for atherosclerosis studies. For atherosclerosis studies, ApoE^−/−^ mice were maintained on WD (42% fat, Tekland, 88137) for 12 weeks.

### Human samples

Patients were recruited through the Heart Transplantation Surgery Clinic at the University of Virginia. This study was carried out in accordance with the recommendations of the National Commission for the Protection of Human Subjects of Biomedical and Behavioral Research, Institutional Review Board for Health Sciences Research (IRB-HSR) at the University of Virginia with written informed consent from all subjects. All patients provided informed written consent prior to participation in this study. The protocol was approved by the IRB-HSR at the University of Virginia. Right coronary artery (RCA) and left anterior descending (LAD) artery and PVAT around RCA and LAD were collected from explanted heart. RCA and LAD arteries were collected for IHC experiments. The stromal vascular fraction was isolated from PVAT around coronary arteries, as described in detail below, for flow cytometry analysis. Peripheral blood mononuclear cells (PBMC) were additionally isolated from whole blood for flow cytometry experiments.

### Flow cytometry

Spleen and bone marrow (BM) cells were harvested and single cell suspensions were prepared as previously described (Srikakulapu et al., 2016). In brief, cell suspension from spleen was prepared using a 70 μm cell strainer and mashing spleen with a syringe plunger, and dissolved in FACS buffer. To isolate BM cells, femur and tibia were collected and flushed with FACS buffer. Spleen and BM samples were re-suspended in erythrocyte lysis buffer and washed. To harvest aorta and PVAT, first, para aortic lymph nodes were carefully removed and then aorta was carefully harvested without having any contamination of PVAT. Aorta and PVAT were collected into 5 ml FACS tubes separately, 2 ml of freshly prepared enzyme cocktail mixture [Collagenase I (450 U/ml) (Sigma), Collagenase XI (125 U/ml) (Sigma), Hyaluronidase I (60 U/ml) (Sigma), DNase (60 U/ml) (Sigma) in PBS with 20 mM HEPES] was added per sample. Samples were chopped into small pieces and then incubated in a shaking incubator at 37°C for 45 min to obtain single cell suspensions. Cells were blocked for Fc receptors by Fc block (CD16/32) for 10 min on ice, and were stained for cell surface markers using fluorescently conjugated antibodies for 30 min on ice. After washing and centrifugation, cells were stained with streptavidin—APC eFluor 780 for 15 min on ice. Cells were washed in PBS and stained with a fixable live/dead stain diluted in PBS for 15 min on ice and then fixed in 2% PFA in PBS for 10 min at room temperature prior to re-suspending in FACS buffer (PBS containing 1% BSA and 0.05% NaN_3_) or sorting buffer (PBS containing 1% BSA) for cell sorting experiments. Flow cytometry antibodies: CD45 (30-F11), CD19 (1D3), B220/CD45R (RA3-6B2), CD5 (53-7.3), CD43 (S7), IgD (11-26.2a), and IgM (II/41, R6-60.2) were purchased from eBioscience, BD Bioscience, and Biolegend. Live/Dead discrimination was determined by LIVE/DEAD fixable yellow staining (Invitrogen) or DAPI (Sigma-Aldrich). Flow cytometry for human PBMCs was performed as published before (Rosenfeld et al., 2015). Human fat (PVAT) was processed as published before (Zimmerlin et al., 2011). In brief, PVAT was placed in PBS supplemented with 5.5 mM glucose and 50 μg/ml gentamicin and processed immediately. One gram of PVAT was minced and digested in 3 ml digestion buffer [PBS + 1% BSA (Gemini) + 2.5 g/L Collagenase II (Worthington)] in a shaking incubator at 37°C for 15 min. After digestion, enzyme reaction was stopped by adding PBS containing 0.1% BSA and 1 mM EDTA. Stromal vascular fraction was then passed through 425 and 180 μm sieves (WS Tyler), and finally through 40 μm filter (BD Falcon). The remaining stromal vascular fraction was stained for flow cytometry. Flow cytometry antibodies: CD45 (2D1), CD20 (L27), CD3e (5KY), CD27 (M-T271), CD43 (84-3C1) were purchased from eBioscience and BD Bioscience. Cells were run on a CyAN ADP (Beckman Coulter) or sorted on an Influx Cell Sorter (Benton-Dickenson). Data were analyzed with FlowJo software (Tree Star inc). All gates were determined using fluorescence minus one (FMO) controls.

### Enzyme-linked immunospot (ELISPOT) assay

Single cell suspensions of aorta, PVAT, spleen and BM were prepared as described above in the flow cytometry section. ELISPOT was performed as previously described (Rosenfeld et al., 2015; Srikakulapu et al., 2016). Sterile MultiScreen IP-Plates (Millipore, MSIPS4510) were used for the assay according to manufacturer's protocol. Wells were coated with unlabeled goat anti-mouse IgM antibody (10 μg/ml; Southern Biotech) or malondialdehyde-modified low density lipoprotein (MDA-LDL) (10 μg/ml) and incubated overnight at 4°C. The following day, antibody solution was decanted, membrane was washed with PBS and then blocked with RPMI 1640+10% FCS for 2 h at 37°C. A suspension of 1 × 10^6^ cells / ml was prepared in ice cold culture media for spleen and BM from which 250,000 cells were plated for each of the sample as starting concentration and then were serially diluted in subsequent wells. For aorta and PVAT samples, total sample resuspended in 250 μl culture media and were used as starting concentration from which serial dilutions in subsequent wells were prepared. The plate was incubated overnight at 37°C in a cell culture incubator (5% CO_2_). Cells were decanted, washed (PBS+0.01% tween-20) and incubated with biotin-labeled goat anti-mouse IgM antibody (1:500 dilution; Southern Biotech) for 2 h in a cell culture incubator. After washing, cells were incubated for 30 min at room temperature in streptavidin alkaline phosphatase (Abcam). Again following washing BCIP/NBT (Gene Tex Inc.) was added and incubated until spots became visible. Each spot on the membrane indicated an antibody secreting cell. Wells were imaged under a dissecting microscope (Zeiss) then spots were counted manually.

### Enface staining

Aortas were isolated as mentioned above in the flow cytometry section. Aortas were opened longitudinally, fixed in 4% formaldehyde, pinned, and stained with Sudan IV (Sigma). Aortas were imaged with a Nikon D70 DSLR camera.

### Histology

Oil red O and Hematoxylin staining was performed as published before (Rosenfeld et al., 2015). Briefly, OCT blocks were prepared for aorta samples with PVAT and without PVAT, sectioned (5 μm) and stored at −80°C. At the time of staining, sections were fixed with 4% formalin for 5 min, 60% isopropanol for 5 min and then stained with Oilred O (O0625, Sigma) for 10 min. After staining, sections were washed briefly in tap water and stained with hematoxylin for 4 min. After washing, sections were mounted with aqueous Vecta mount (H-5501, Vector). Hematoxylin and eosin staining was performed in human coronary artery sections. First, slides were de-paraffinized and hydrated through two changes of xylene, 100, 95% ethanol, and one change of 70% ethanol by incubating slides for 5 min in each change. Slides were rinsed with two changes of distilled tap water and stained with Hematoxylin for 5 min. After rinsing, bluing was performed in Ammonia water. After rinsing, slides were incubated in 70% ethanol and stained with Eosin for 10 min. Slides were dehydrated through three changes of 100% ethanol, followed by two changes of Xylene, 5 min each. Slides were mounted with aqueous clear mounting media. Images were taken using Olympus Hi-Mag microscope.

### Multiplex immuno histo chemistry (IHC)

The Opal Multiplex Manual IHC Kit (PerkinElemer, Waltham, MA) was used to perform IHC staining. Tonsil and normal lymph node specimens were used as positive and negative staining controls. Single stained slides were also generated for use in spectral unmixing. Staining was performed according to the manufacturer's protocol; however, slides were allowed to cool for 30 min at room temperature post microwaving in Antigen Retrieval (AR) buffer. To facilitate continuation of staining, slides were stored overnight in AR buffer at 4°C. Staining sequence and antibodies: AR9—CD8 (dilution 1:500, Dako, Santa Clara, CA)—Opal540, AR6—CD20 (1:1000, Dako)—Opal520, AR6—PNAD (1:1000, BD Biosciences, Franklin Lakes, NJ)—Opal620 and AR6—DAPI for nuclear staining. Slides were mounted used Prolong diamond antifade (Life Technologies, Carlsbad, CA). Pictures were taken with Vectra microscope (PerkinElemer).

### Statistics

Student's *t*-test was used for analyzing data with normal distribution and equal variance. For data sets with unequal variance, *t*-test with Welch's correction was used. Repeated measures one way ANOVA with Bonferroni's multiple comparison post-test was used to compare multiple groups. Results are displayed containing all replicated experiments, and values shown are mean ± SEM. Data were analyzed using Prism 5 (GraphPad Software, Inc).

## Results

### PVAT is the predominant source of aortic-associated B lymphocytes

**Figure 1:**
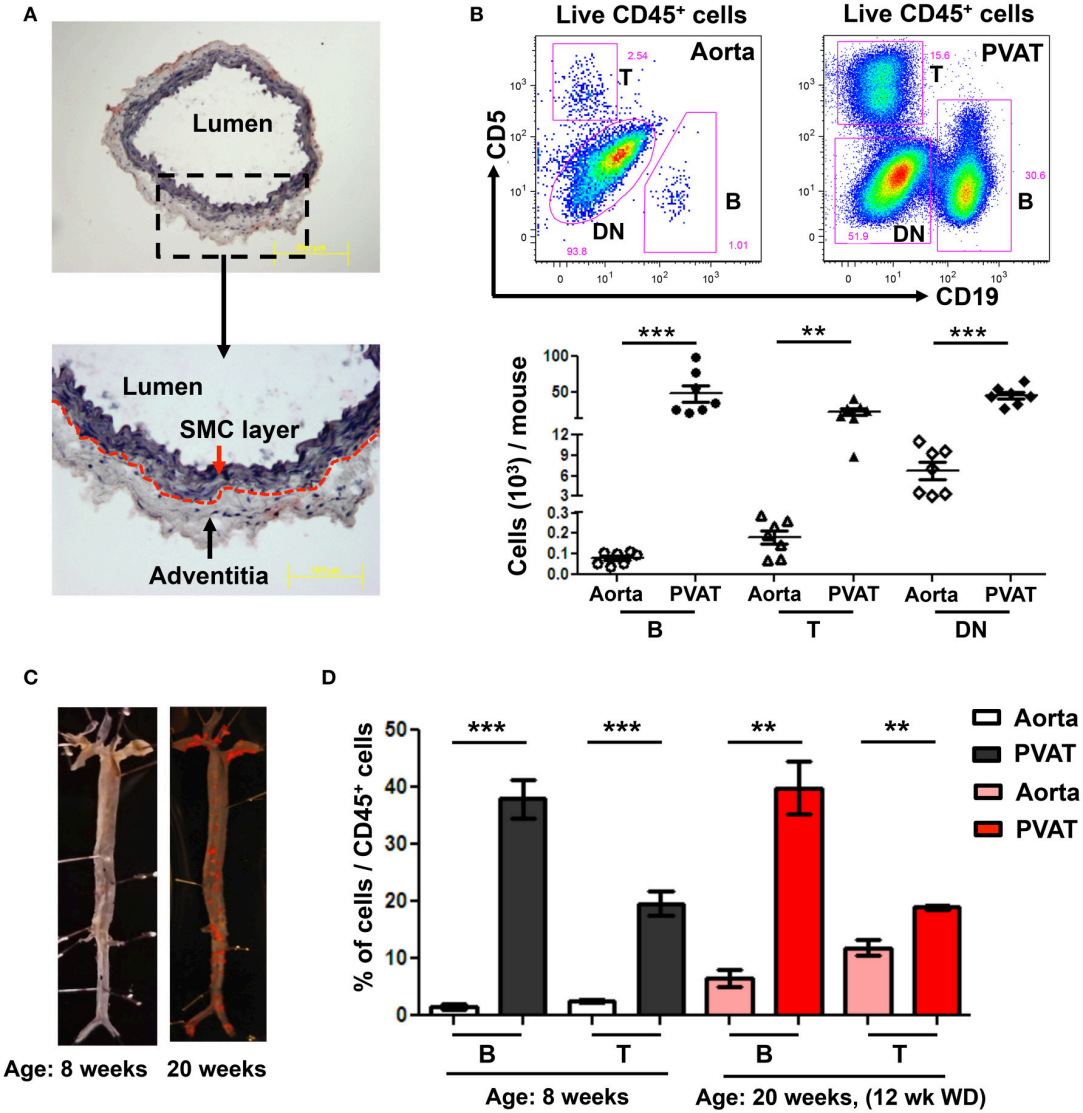
High numbers of B and T cells are harbored in perivascular adipose tissue (PVAT). **(A)** Aorta (without PVAT) of young ApoE^−/−^ mice was carefully dissected, stained with OilRedO and Hematoxylin. Dotted line represents the border between smooth muscle cell (SMC) and adventitial layers. **(B)** CD19^+^ B cells, CD5^+^ T cells and CD5 and CD19 double negative (DN) non T and B cells were gated from total live CD45^+^ cells and these B, T and DN subsets were quantified in aorta and PVAT of 8–10 weeks old ApoE^−/−^ mice fed normal Chow diet by flow cytometry. **(C)** Representative Sudan IV staining of aortas collected from Chow-fed ApoE^−/−^ mice at 8 weeks and 20 weeks (maintained on Western diet (WD) for the last 12 weeks) of age. **(D)** Quantification of the percent of leukocytes (CD45^+^) in the aorta and PVAT of these 8 weeks (*n* = 7) and 20 weeks (*n* = 3) old ApoE^−/−^ mice that were CD19^+^ B cells and CD5^+^ T cells. Results are mean ± SEM, unpaired student *t*-test was performed (^**^p < 0.01, ^***^p < 0.001).

To determine the predominant location of aortic-associated B cells in atherosclerosis-prone mice at homeostasis, flow cytometry was performed in young ApoE^−/−^ mice (8 weeks old) fed normal Chow diet. Aorta, from the arch to the iliac bifurcation was carefully dissected to retain the adventitia excluding PVAT. Oil-red O/ hematoxylin staining of aorta cross sections confirmed that the dissected aorta retained the adventitia but no PVAT (Figure 1A). Flow cytometry revealed greater numbers of CD19^+^ B cells, CD5^+^ T cells and double negative cells (DN: CD19^−^ CD5^−^) in the PVAT compared to the aorta (Figure 1B). Lymphocytes (B and T) made up a greater percentage of the total leukocytes (CD45^+^) in the PVAT compared to the aorta. In contrast, DN cells made up a greater percentage of CD45^+^ cells in the aorta compared to the PVAT, although PVAT still contained large numbers of DN cells (Figure 1B). Next, to determine the effect of WD and atherosclerosis development on B and T cells in aorta and PVAT, 8 week old ApoE^−/−^ mice were fed 12 weeks of WD and flow cytometry for B and T cells was performed. Sudan IV staining for atherosclerosis lesions in 8 and 20 weeks old mice confirmed atherosclerosis development in the ApoE^−/−^ mice fed 12 weeks of WD but not in 8 week old Chow fed ApoE^−/−^ mice (Figure 1C). The percentage of CD45^+^ cells that are B and T cells was significantly higher in the PVAT than in the aorta in both Chow fed and 12 weeks WD fed mice (Figure 1D).

### B cells reside in human coronary artery PVAT

**Figure 2:**
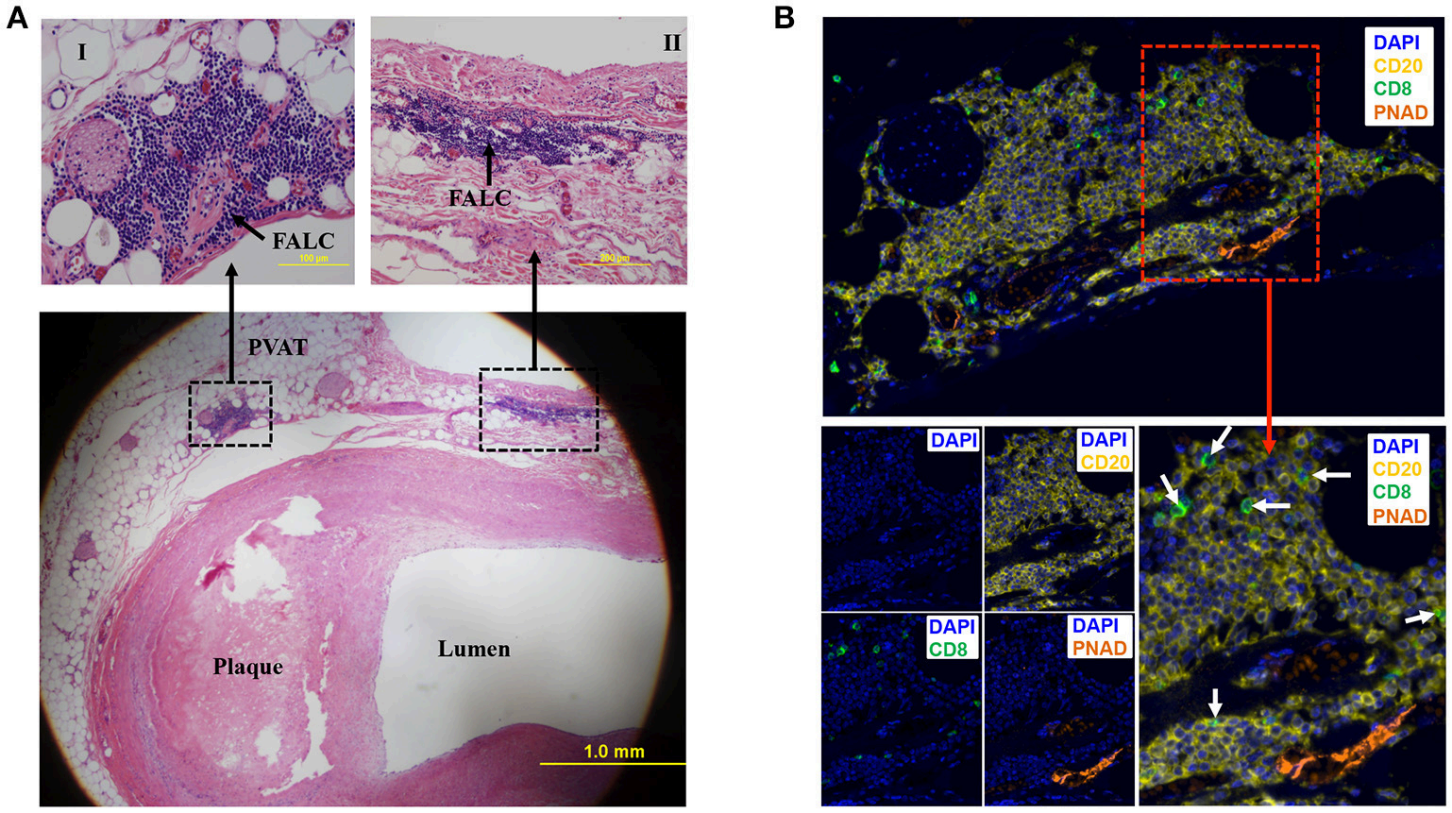
Fat associated lymphoid clusters (FALCs) in human coronary artery PVAT. **(A)** Sections of diseased human coronary artery including PVAT (*n* = 1) were stained with hematoxylin and eosin. FALCs were identified in the PVAT adjacent to coronary artery. High magnification of FALCs (I and II) regions are indicated in dotted box. **(B)** Consecutive sections of hematoxylin stained slides were used for multiplex IHC staining (*n* = 1). CD20^+^ B cells, CD8^+^ T cells and PNAD^+^ high endothelial venules were observed in FALC. CD8^+^ T cells were marked with white arrows (merged lower left picture). DAPI stains for nuclear DNA.

To determine if human PVAT contains FALCs, coronary arteries from human hearts explanted at the time of heart transplantation were sectioned and stained with eosin and hematoxylin. Microscopic examination of these sections revealed that in addition to scattered lymphocytes throughout the PVAT, the PVAT also contained FALCs in the PVAT close to the diseased coronary artery (Figure 2A). Next, we performed multiplex IHC to determine what type of cells and structures are located in these FALCs. The majority of the immune cells in human FALCs were CD20^+^ B cells (Figure 2B). These FALCs also harbored few numbers of CD8^+^ T cells (Figure 2B), FoxP3^+^ regulatory T cells and proliferating Ki67^+^ cells (data not shown). In addition, FALCs in human coronary PVAT contained structures of PNAD^+^ high endothelial venules (HEV) (Figure 2B), which are important for lymphocyte recruitment into lymphoid tissues (Girard and Springer, 1995).

**Figure 3:**
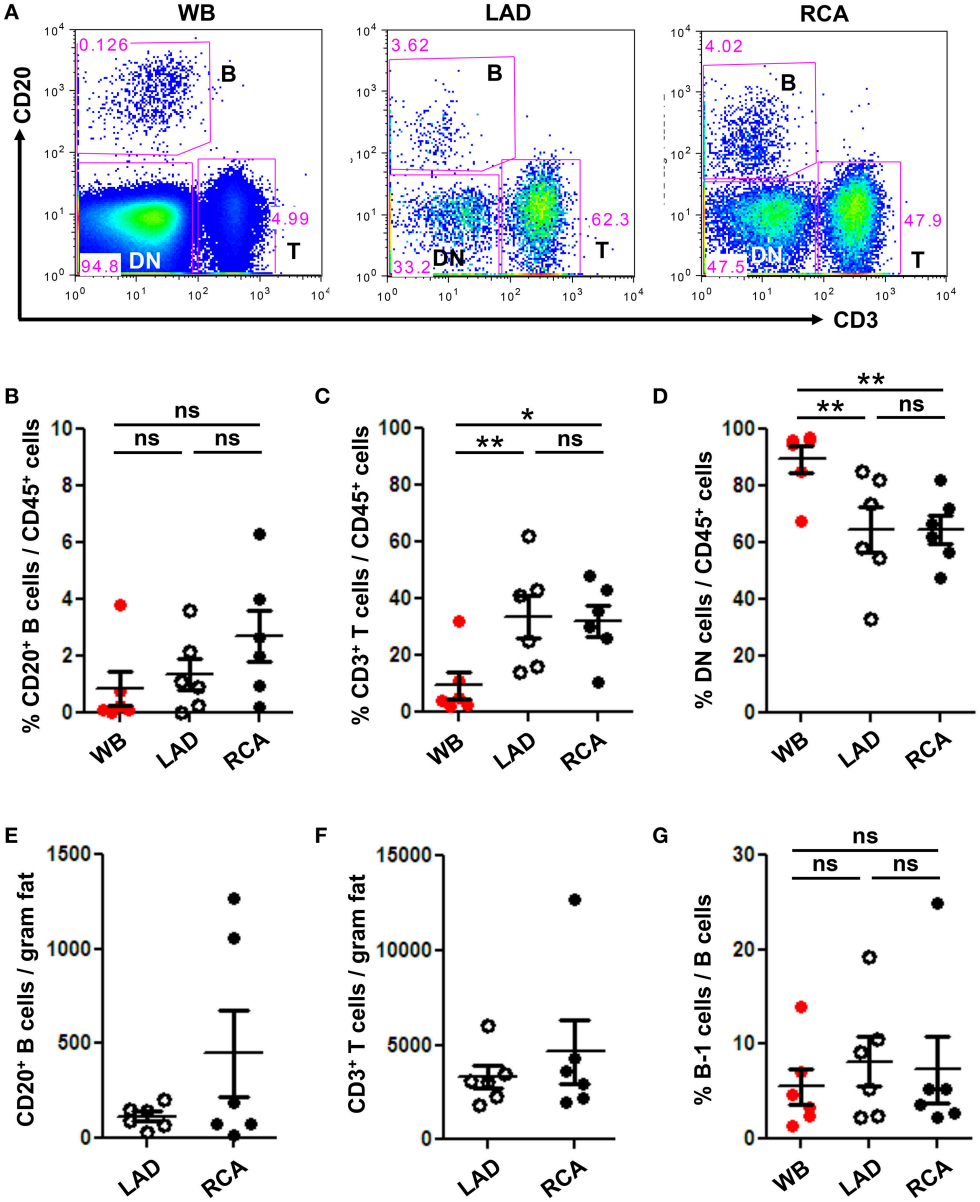
B and T cells reside in human coronary artery PVAT. Coronary artery PVAT from human hearts (*n* = 6) explanted at the time of heart transplantation was analyzed by flow cytometry. **(A)** Gating strategy of B cells (CD20^+^), T cells (CD3^+^), and DN (CD45^+^ non B and T cells) in whole blood (WB), PVAT near LAD, and RCA for flow cytometry. Percentages of **(B)** B cells, **(C)** T cells, and **(D)** DN cells from total CD45^+^ cells were quantified in PVAT from the LAD and RCA, and WB as a comparator. Total number of **(E)** B cells and **(F)** T cells per gram adipose tissue was quantified. **(G)** Quantification of the percentage of B cells that were B-1 (CD20^+^ CD27^+^ CD43^+^). Results are mean ± SEM, unpaired student *t*-test was performed. Repeated measures one way ANOVA with Bonferroni's multiple comparison post-test was used to compare multiple groups (^*^P < 0.05, ^**^P < 0.01).

To quantify immune cell subtypes in human coronary artery PVAT, a large segment of PVAT from the coronary arteries of human hearts explanted at the time of heart transplantation was harvested and analyzed by flow cytometry. Blood was analyzed as a comparator. Total CD20^+^ B cells, CD3^+^ T cells and DN (CD20^−^ CD3^−^) non B and T cells were gated from total CD45^+^ live cells (Figure 3A). Results demonstrated that PVAT near coronary arteries contain B cells, T cells, and DN cells (Figures 3B–D). This finding is not due to blood contamination as the percentage of T cells is actually lower and the percentage of DN cells is higher in the WB (Figures 3C,D). Quantification of lymphocyte numbers (B and T cells) per gram of fat revealed equivalent numbers in PVAT from LAD and RCA (Figures 3E,F). In contrast to murine aortic PVAT that was dissected down to the region directly abutting the adventitia (Figures 1B,D), the majority of the lymphocytes in the human PVAT removed without care to include the peri-adventitial area where FALC are present were T cells (Figures 3C,F). B cells were also present and of these nearly 7–8% were B-1 cells (Figure 3G).

### B-1 cells are enriched in PVAT and secrete IgM locally

**Figure 4:**
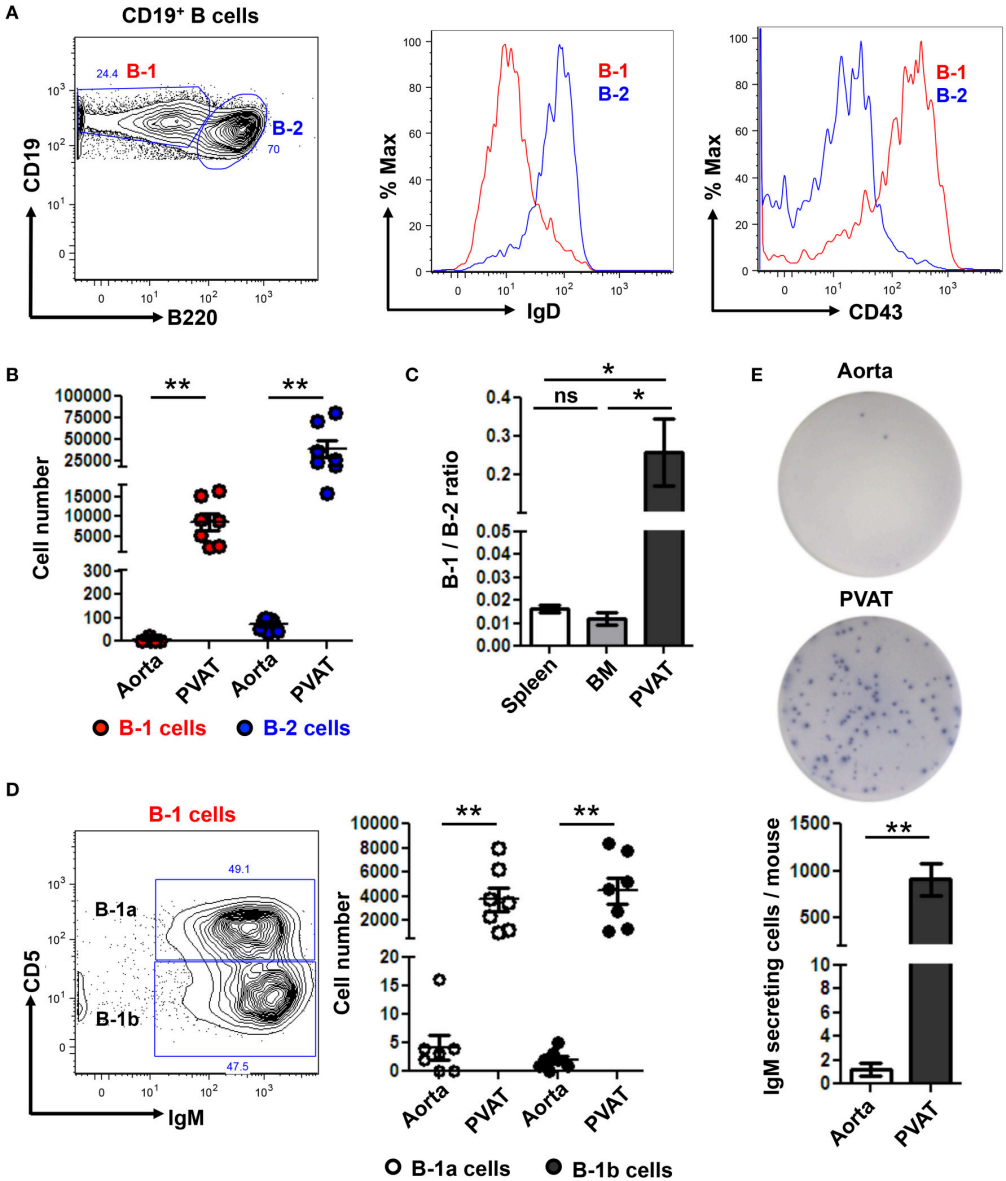
IgM secreting B-1 cells reside in PVAT. **(A)** Gating strategy of B-1 (CD19^+^, B220^lo-*mid*^) and B2 (CD19^+^, B220^hi^) cells. B-1 (IgD^−^ CD43^+^) and B-2 (IgD^+^ CD43^−^) cells were further confirmed based on surface expression of IgD and CD43. **(B)** Quantification of total numbers of B-1 and B-2 cells in the aorta and PVAT. **(C)** Comparative ratio of B-1 to B-2 cells in spleen, bone marrow (BM) and PVAT. **(D)** B-1a and B-1b cells were gated from total B-1 cells and absolute numbers were quantified in aorta and PVAT of young ApoE^−/−^ mice. **(E)** IgM antibody production was measured by ELISPOT in aorta and PVAT of Chow diet fed young ApoE^−/−^ mice (*n* = 7) (representative plate and quantitation). Results are mean ± SEM, unpaired student *t*-test was performed. Repeated measures one way ANOVA with Bonferroni's multiple comparison post-test was used to compare multiple groups (^*^p < 0.05, ^**^p < 0.01).

We returned to our murine model where we are able to carefully dissect all the PVAT down to the adventitial interface (Figure 1A) to further subset the B cells in the aorta and PVAT and to determine if IgM can be locally produced. Aorta and PVAT were carefully separated and flow cytometry to identify B cell subtypes was performed. B-1 (CD19^+^ B220^lo-*mid*^) and B-2 (CD19^+^ B220^hi^) cells were confirmed based on surface expression of IgD and CD43 (Figure 4A). Significantly greater numbers of both B-1 and B-2 cells were present in PVAT compared to the aorta (Figure 4B). Notably, while B-2 cells are the predominant B cell subtype in the spleen and bone marrow (Srikakulapu and McNamara, 2017), there was a >10-fold higher B-1/B-2 ratio in PVAT compared to these lymphoid organs (Figure 4C). In mice, B-1 cells are divided into B-1a and B-1b based on surface expression of CD5 (Figure 4D). Flow cytometry revealed small numbers of B-1a and B-1b cells in the aorta. In contrast, significantly higher numbers of both subsets were present in the PVAT (Figure 4D). To determine whether these B cells are capable of antibody production in aorta and PVAT, ELISPOT experiments were performed. Very low numbers of IgM secreting cells were in aorta. In contrast, there were abundant IgM secreting cells in PVAT (Figure 4E).

### B cell specific Id3 deficiency increases B-1b cell numbers and MDA-LDL specific IgM production in PVAT

**Figure 5:**
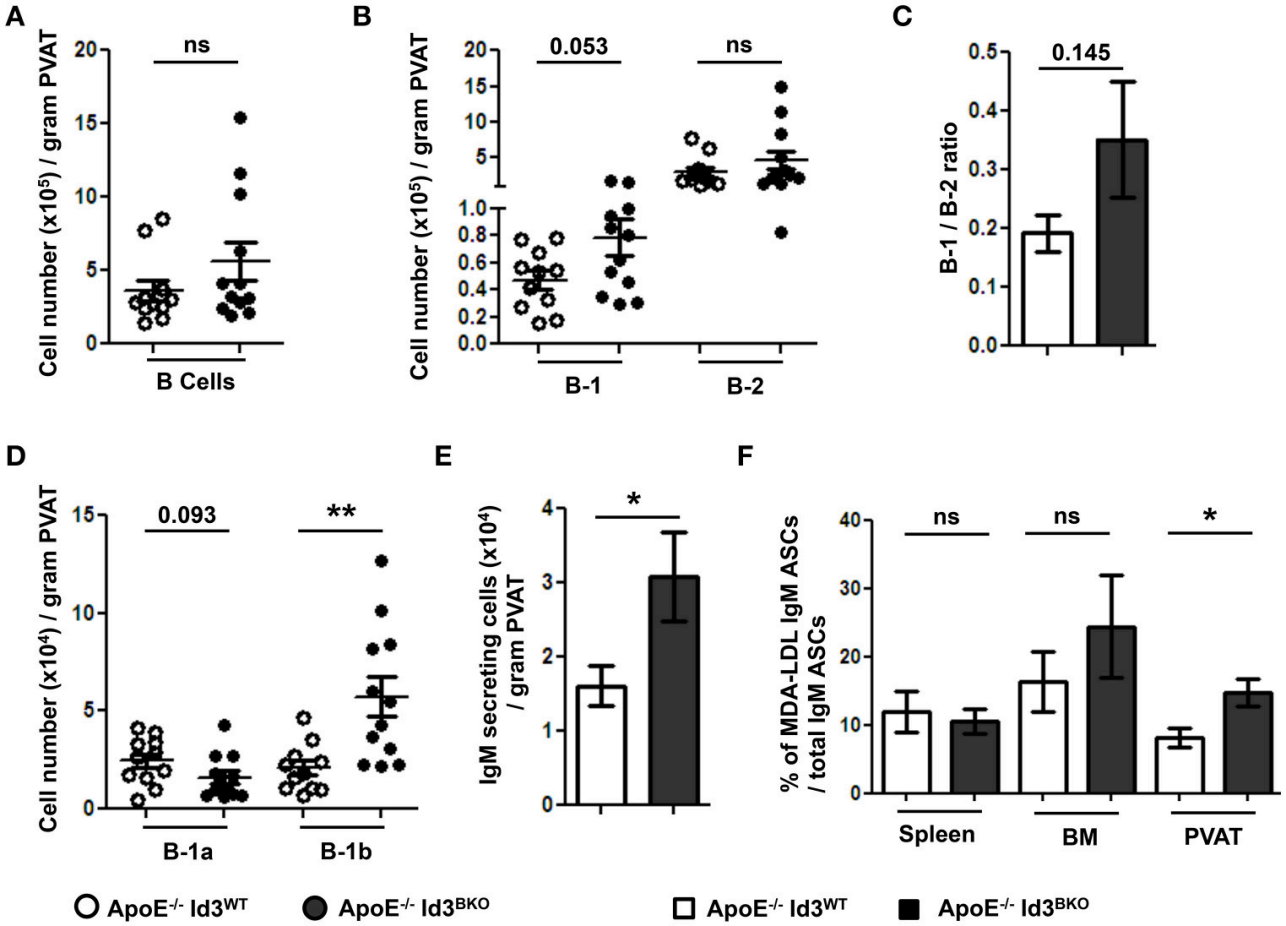
B-1b cells and IgM secreting B cells are increased in the PVAT of mice with B cell specific deletion of Id3 (ApoE^−/−^Id3^BKO^). Flow cytometry quantification in the PVAT of 8 weeks old ApoE^−/−^Id3^WT^ (*n* = 11) and ApoE^−/−^Id3^BKO^ (*n* = 12) mice for **(A)** total CD19^+^ B cells, **(B)** B-1 and B-2 cells, **(C)** B-1/B-2 ratio, **(D)** B-1a and B-1b cells, and **(E)** ELISPOT for total IgM secreting cells. **(F)** Percentage of malondialdehyde modified low density lipoprotein (MDA-LDL) specific IgM secreting cells in spleen, BM and PVAT of ApoE^−/−^Id3^WT^ (*n* = 5) and ApoE^−/−^Id3^BKO^ (*n* = 4–5) mice as measured by ELISPOT. Results are mean ± SEM, unpaired student *t*-test was performed (^*^p < 0.05, ^**^p < 0.01).

Next, to determine the effect of B cell specific Id3 deficiency on B cells in PVAT, B cell subsets in PVAT of young (8–10 weeks) ApoE^−/−^Id3^WT^ and ApoE^−/−^Id3^BKO^ mice were quantified by flow cytometry. There was no difference in total B cell and B-2 cell numbers. However, there was a trending increase in number of B-1 cells as well as B-1/B-2 ratio in the PVAT of ApoE^−/−^Id3^BKO^ mice compared to ApoE^−/−^Id3^WT^ control mice (Figures 5A–C). Notably, while B-1a cell numbers trended to be lower, B-1b cells were significantly greater in PVAT of ApoE^−/−^Id3^BKO^ mice compared to ApoE^−/−^Id3^WT^ control group mice (Figure 5D). ELISPOT data demonstrated increased numbers of IgM secreting cells in the PVAT of ApoE^−/−^Id3^BKO^ compared to ApoE^−/−^Id3^WT^ mice (Figure 5E). To determine how much of this IgM recognized specific OSE, ELISPOT for MDA-LDL-specific IgM was performed in spleen, BM and PVAT of ApoE^−/−^Id3^WT^ and ApoE^−/−^Id3^BKO^ mice. Interestingly, there was a significantly higher percentage of MDA-LDL-specific IgM observed in the PVAT but not in spleen and BM of ApoE^−/−^Id3^BKO^ mice compared to ApoE^−/−^Id3^WT^ control mice (Figure 5F), suggesting that local MDA-LDL from the aorta or PVAT in the context of greater B-1b cell numbers may lead to enhanced production of MDA-LDL specific IgM.

## Discussion

PVAT inflammation promotes atherosclerosis in the underlying vessel through “outside-in” signaling (Brown et al., 2014; Omar et al., 2014; Konaniah et al., 2017). Adipocyte-derived MCP-1 (Manka et al., 2014) and low-density lipoprotein receptor-related protein-1 (Konaniah et al., 2017) are two of the factors that have been implicated in promoting PVAT inflammation. However, immune cell composition of the PVAT at homeostasis and in response to atherosclerosis and molecular mechanisms that regulate the proportion of pro- and anti-inflammatory immune cell subsets are poorly understood. Here, we provide the first evidence that the immune cell composition of the PVAT of young (8–10 weeks), Chow fed ApoE^−/−^ mice is distinct from that of the aorta, with PVAT harboring higher numbers of leukocytes, notably B and T lymphocytes. In contrast, aorta primarily contains CD45^+^ non B and T cells at steady state. Previous studies have demonstrated that macrophages are abundant in the CD45^+^ non B and T cell compartment in the aorta of Chow fed ApoE^−/−^ mice (Galkina et al., 2006). Recent studies report that resident macrophages (CD11b^+^F4/80^+^CD115^+^Lyve-1^+^) in the aorta are derived from yolk sac and fetal liver during development and these resident macrophages have self-renewal capacity and maintain tissue homeostasis in steady state. In response to inflammatory signals, inflammatory macrophages (CD11b^+^F4/80^+^CD115^−^Lyve-1^−^) from BM, are recruited into the aorta (Ensan et al., 2016). Consistent with this, WD fed ApoE^−/−^ mice have a marked increase in activated macrophages in the aorta (Galkina et al., 2006). There is also an increase in B and T cells in the aorta of WD fed mice (Galkina et al., 2006). However, the predominance of lymphocytes in the PVAT compared to aorta persisted after WD (Figure 1D), suggesting that PVAT lymphocytes play key roles in both tissue homeostasis and in regulating inflammation due to lipid deposition in the artery wall. Whether lymphocytes in the aorta are recruited from the PVAT and whether lesion immune cell accumulation is regulated by lymphocytes in the PVAT remain important unanswered questions.

Notably, in contrast to the aorta, B cells outnumber T cells in PVAT of ApoE^−/−^ mice. As such, defining B cell subsets in the PVAT may provide important insights into PVAT regulation of inflammation. B cells have been implicated in regulating visceral adipose tissue inflammation and its metabolic consequences. B-2 cells worsen insulin sensitivity through their production of IgG (Winer et al., 2011) while B-1 derived natural IgM and regulatory B cell (B_regs_)-derived anti-inflammatory cytokine interleukin −10 (IL-10), attenuate adipose tissue inflammation, glucose intolerance, and improve metabolic syndrome in diet induced obese mice and humans (Nishimura et al., 2013; Harmon et al., 2016). Yet, B cell subset composition of PVAT is relatively unknown. In the present study, we provide the first evidence that, in contrast to other sites that harbor significant B cell accumulation (spleen and BM), the PVAT contains a significantly higher B-1/B-2 ratio suggesting that B cells in PVAT may have anti-inflammatory effects. Of these B-1 cells, there are equal numbers of B-1a and B-1b cells in the PVAT. Both of these B-1 subtypes have been shown to produce atheroprotective IgM and attenuate atherosclerosis in mice (Kyaw et al., 2011; Rosenfeld et al., 2015). Consistent with prior studies that B-1 cells are the predominant source of IgM (Corte-Real et al., 2009; Holodick et al., 2010; Choi et al., 2012), equivalent numbers of cultured B-1 cells from spleen, BM (sites considered to be the main source of B-1 cell-derived IgM) and PVAT produced substantially more IgM than B-2 cells from these same tissues (data not shown).

Previous studies have demonstrated that PVAT differs between species and anatomic location. In rodents, the thoracic aorta is predominantly surrounded by brown adipose tissue, whereas the abdominal aorta is surrounded by a mixture of white and brown adipose tissue (Brown et al., 2014). The expression of genes encoding inflammatory cytokines and infiltrated immune cell markers are greater in PVAT near abdominal aorta than thoracic aorta of rats (Padilla et al., 2013). In addition, our group has reported that radiolabeled splenic B cells, which are predominantly B-2 cells, adoptively transferred to B cell deficient mice were preferentially recruited into the aortic arch and abdominal aortic region. However, no/very low numbers of B cells were recruited into the thoracic aorta region (Doran et al., 2012). Altogether, these reports suggest that PVAT at different parts of the aorta have different immune cell compositions. Future studies are needed to provide more insights into aortic region-specific differences in immune cell composition of PVAT and its impact on region-specific aortic vascular diseases.

There may also be important differences between coronary and aortic PVAT as the adipocytes in these regions are thought to derive from unique embryologic origins and may differ metabolically (Aldiss et al., 2017). This is important as inflammation of the PVAT around coronary arteries has been linked to coronary artery disease pathophysiology. In humans, PVAT thickness around coronary arteries has been associated with coronary artery calcification, cardiovascular risk factors (de Vos et al., 2008), the degree of plaque burden (Iacobellis and Willens, 2009), and stenosis (Verhagen et al., 2012). The number of macrophages in PVAT has been related to the size and characteristics of the atherosclerotic plaque such as, lipid core, calcification, collagen, and smooth muscle cell content, and to the degree of plaque infiltration by macrophages and lymphocytes (Konishi et al., 2010; Verhagen et al., 2012). In addition, the PVAT near coronary arteries and epicardial adipose tissue has been reported to show high levels of inflammatory cytokines (Mazurek et al., 2003; Chatterjee et al., 2009) and infiltration of leukocytes (Konishi et al., 2010), particularly macrophages and T lymphocytes (Mazurek et al., 2003; Hirata et al., 2011). As such, strategies to modify PVAT inflammation may impact on underlying CVD. This underscores the importance of characterizing human coronary artery PVAT. Our findings demonstrated that, in contrast to our murine aortic findings, T cells outnumbered B cells in the human coronary PVAT analyzed by flow cytometry. Whether this was due to differences in coronary vs. aortic PVAT or murine vs. human PVAT was difficult for us to resolve directly as murine coronary PVAT yields insufficient cells for analysis by flow cytometry and we were unable to take aortic samples from our live subjects donating their explanted hearts at the time of heart transplantation for our human coronary PVAT analysis. Our IHC staining of the human coronary PVAT may provide one explanation to resolve this discrepancy. Consistent with our murine aorta flow cytometry, the predominant lymphocyte that aggregates in FALCs in close proximity to the coronary artery are B cells. It is notable that the PVAT used for the human flow cytometry was not as closely associated with the artery as the FALC. As such, while flow cytometry confirmed that B cells including B-1 were present in human PVAT, future studies utilizing flow cytometry will be needed to analyze the immune composition of human PVAT in close proximity compared to more distant to the coronary artery to help resolve this issue. In addition, further studies to understand site-specific effects of B-1 cells on PVAT inflammation are needed.

We had previously shown that B-1 cell-derived IgM inhibited inflammatory cytokine production by M1 macrophages in visceral adipose tissue and promoted insulin sensitivity in mice (Harmon et al., 2016). Furthermore, B-1 cells and IgM to oxidation-specific epitopes (OSE) on LDL were found in human omental adipose tissue and there was a correlation between omental production and plasma levels of IgM to OSE. Moreover, levels of IgM to OSE inversely correlated with plasma levels of the inflammatory cytokine MCP-1, suggesting that homeostatic immune mechanisms in adipose tissue may dampen both local and systemic inflammatory responses (Harmon et al., 2016). As plasma levels of IgM to OSE are inversely associated with coronary artery disease in humans (Tsimikas et al., 2007, 2012), further studies are needed to determine if PVAT B-1 cell production of IgM to OSE can reduce PVAT inflammation and the atherosclerosis lesion underlying it.

Earlier study from our lab demonstrated that C57BL6 mice with B cell specific Id3 deficiency had greater visceral adipose tissue B-1b cells and IgM production and less adipose tissue inflammation compared to WT littermate controls (Harmon et al., 2016). Here, we demonstrate for the first time that Id3 also regulates the number of B-1b cells in the PVAT. Consistent with the higher number of B-1b cells in ApoE^−/−^Id3^BKO^ compared to control group mice, we demonstrate that ApoE^−/−^Id3^BKO^ mice have significantly greater amounts of IgM in the PVAT. Notably, not only was the amount of total IgM higher, the percentage of IgM that was specific for MDA-LDL was greater in the PVAT of ApoE^−/−^Id3^BKO^ mice compared to littermate controls. Previous studies have demonstrated that circulatory IgM but not IgG to MDA-LDL negatively correlates with angiographically determined coronary artery disease and CV events in humans (Tsimikas et al., 2007, 2012) suggesting a protective role of IgM to MDA-LDL in atherosclerosis. The fact that MDA-LDL specific IgM was greater in PVAT of ApoE^−/−^Id3^BKO^ mice, but not in other major IgM producing sites such as, BM and spleen, raises the interesting possibility that the IgM responses in PVAT may be stimulated by local sources of OSE. While the source of modified LDL in atherosclerotic lesions had been thought to come from the circulation, Uchida et al, recently provided data to support PVAT as a source of OxLDL in human coronary lesions (Uchida et al., 2016). Additional studies are needed to determine if PVAT may be an important source of inflammation stimulating OSE.

Understanding immune modulation of PVAT in mice may provide novel strategies to limit atherosclerosis development in humans. However, direct evaluation of PVAT surrounding human coronary arteries is important for determining if these murine findings may be relevant to human disease. Recent studies have shed light on the role of FALCs in adipose tissue. FALCs develop in both steady state and inflammatory condition (Benezech et al., 2015). It has been shown that, B cells in FALCs secrete IgM and attenuate local inflammation (Jackson-Jones et al., 2016). Recent studies have demonstrated that innate lymphoid cell type-2 derived IL-13 and IL-5 cytokines in PVAT are important to maintain atheroprotective IgM producing B-1 cells in PVAT and attenuate diet induced atherosclerosis (Perry et al., 2013; Newland et al., 2017). However, it is not known whether B and T cells are present in specialized structures like FALCs in the young murine PVAT or whether they are scattered in the entire tissue. The present study demonstrates that PVAT adjacent to diseased coronary artery in humans contains FALCs. Similar to FALCs in mice (Benezech et al., 2015; Newland et al., 2017), FALCs in human PVAT harbors abundant B cells.

In summary, our results provide novel evidence that atheroprotective B-1 cells accumulate in PVAT and secrete natural IgM, particularly MDA-LDL specific IgM. Based on our murine findings that B-1b cells in the adipose tissue regulate M-1 macrophage inflammation (Harmon et al., 2016), we hypothesize that B-1 cells in the PVAT can modulate local inflammation and potentially atherosclerosis development. Further studies are needed to test this novel hypothesis. These studies will be important to do given findings that B-1 cells reside in human PVAT and B cell-rich FALC are closely associated with coronary arteries. These findings may lead to novel strategies targeting PVAT to limit atherosclerosis development.

## Author contributions

PS: designed and performed the experiments, acquired and analyzed the data, prepared figures, and wrote the manuscript. AU, SR, MM, CM, AH, and IM performed the experiments. GA, ML, and AT: provided human samples. CAM: designed the experiments and wrote the manuscript.

### Conflict of interest statement

The authors declare that the research was conducted in the absence of any commercial or financial relationships that could be construed as a potential conflict of interest.
